# Estimating growth and photosynthetic properties of wheat grown in simulated saline field conditions using hyperspectral reflectance sensing and multivariate analysis

**DOI:** 10.1038/s41598-019-52802-5

**Published:** 2019-11-11

**Authors:** Salah El-Hendawy, Nasser Al-Suhaibani, Majed Alotaibi, Wael Hassan, Salah Elsayed, Muhammad Usman Tahir, Ahmed Ibrahim Mohamed, Urs Schmidhalter

**Affiliations:** 1 Department of Plant Production, College of Food and Agriculture Sciences, King Saud University, P.O. Box 2460, 11451 Riyadh, Saudi Arabia; 2 Department of Agronomy, Faculty of Agriculture, Suez Canal University, Ismailia, 41522 Egypt; 3 Department of Agricultural Botany, Faculty of Agriculture, Suez Canal University, Ismailia, 41522 Egypt; 4 Department of Biology, College of Science and Humanities at Quwayiah, Shaqra University, Riyadh, 11961 Saudi Arabia; 5 Evaluation of Natural Resources Department, Environmental Studies and Research Institute, University of Sadat City, Menoufia, 32897 Egypt; 6 Department of Soil and Water, Faculty of Agriculture, Suez Canal University, Ismailia, 41522 Egypt; 7 Department of Plant Science, Chair of Plant Nutrition, Technical University of Munich, Freising, Germany

**Keywords:** Abiotic, Plant physiology

## Abstract

The timely estimation of growth and photosynthetic-related traits in an easy and nondestructive manner using hyperspectral data will become imperative for addressing the challenges of environmental stresses inherent to the agricultural sector in arid conditions. However, the handling and analysis of these data by exploiting the full spectrum remains the determining factor for refining the estimation of crop variables. The main objective of this study was to estimate growth and traits underpinning photosynthetic efficiency of two wheat cultivars grown under simulated saline field conditions and exposed to three salinity levels using hyperspectral reflectance information from 350–2500 nm obtained at two years. Partial least squares regression (PLSR) based on the full spectrum was applied to develop predictive models for estimating the measured parameters in different conditions (salinity levels, cultivars, and years). Variable importance in projection (VIP) of PLSR in combination with multiple linear regression (MLR) was implemented to identify important waveband regions and influential wavelengths related to the measured parameters. The results showed that the PLSR models exhibited moderate to high coefficients of determination (R^2^) in both the calibration and validation datasets (0.30–0.95), but that this range of R^2^ values depended on parameters and conditions. The PLSR models based on the full spectrum accurately and robustly predicted three of four parameters across all conditions. Based on the combination of PLSR-VIP and MLR analysis, the wavelengths selected within the visible (VIS), red-edge, and middle near-infrared (NIR) wavebands were the most sensitive to all parameters in all conditions, whereas those selected within the shortwave infrared (SWIR) waveband were effective for some parameters in particular conditions. Overall, these results indicated that the PLSR analysis and band selection techniques can offer a rapid and nondestructive alternative approach to accurately estimate growth- and photosynthetic-related trait responses to salinity stress.

## Introduction

Salinity and the decreasing availability of freshwater are major factors restricting the productivity of agricultural crops in arid and semiarid regions. In addition, the lack of fresh water available to the agriculture sector in such regions has necessitated a substantial increase in the use of saline water resources. This has exacerbated the adverse effects of salinity on crop productivity. To enable the application of suitable protective measures, it is necessary to conduct studies that focus on the timely detection of the extent and magnitude of the impacts of salinity stress on the plant physiological status.

There are numerous morphophysiological parameters such as dry matter accumulation and photosynthetic properties [photosynthesis (*Pn*) and transpiration (*E*) rates, and stomatal conductance (*Gs*)] that are useful in the detection of a plant’s physiological status under salinity stress. These parameters provide valuable information that can be used to evaluate the salt tolerance of genotypes in a breeding program, understand the mechanisms of salt tolerance, and support the growth and production of crops under salinity stress conditions through the application of appropriate agronomic practices^1,2^. However, tracking the dynamic changes of photosynthetic properties or the real-time detection of dry matter accumulation using destructive plant sampling methods and several observations are generally time-consuming, expensive, and laborious. In particular, although changes in the photosynthetic properties can be detected in nondestructive manner using a gas exchange system device, this device detects photosynthetic properties based on a single effective leaf and disregards the vertical variability in photosynthetic efficiency within the plant canopy and is time-consuming and hence limited to few measurements in a given time^3,4^.

A reasonable solution to address these issues is tracking the changes of these parameters using narrow-band visible (VIS)-to-shortwave infrared (SWIR) hyperspectral sensing. This allows to simultaneously monitor diverse and multiple specific alterations that could be induced by osmotic and ionic stresses of salinity such as changes in the internal leaf structure, plant water status, photosynthetic pigments and nutrient contents, photosynthetic potential, biomass accumulation, chlorophyll fluorescence, and more^5–10^. Consequently, alterations in these specific variables result in substantial variations in the absorption of specific wavebands in the VIS-SWIR domains of the spectrum. For example, changes in photosynthetic pigments and their functioning impact the reflectance of the 680 and 740 nm wavelengths^11^. El-Hendawy *et al*.^12^ reported that changes in leaf water relations and ion content in wheat plants grown under salinity stress conditions influence the spectral reflectance in the VIS (434–488 nm and 503–632 nm), red-edge (701–743 nm), near-infrared (NIR; 1100–1300 nm), and SWIR (1706–1898 nm) regions.

Tilling *et al*.^13^ also reported that changes in the internal leaf structure owing to decreases in water content resulted in a significant change in the spectral reflectance in the regions of red edge (680–740 nm) and NIR (740–940 nm). The changes in *Gs* induced by water stress impacted the spectral reflectance in the range of 400–720 nm of the spectrum^14^. Therefore, these close relationships between the specific canopy variables and specific wavebands make this tool more effective than traditional tools for estimating the biomass and photosynthetic properties of plants in a rapid, cheap, and nondestructive manner.

Generally, in order to indirectly assess different plant parameters through their canopy reflectance signatures, the majority of the previous studies focused on the significance of relationships between the plant parameters and specific spectral reflectance indices (SRIs). However, most SRIs use very few principal wavebands (one, two, or three specific wavelengths) and discard the majority of other wavelengths in the full VIS-SWIR spectrum. These very limited numbers of wavelengths results in SRIs that tend to be less efficient and inconsistent for assessing plant parameters across different growing environmental conditions, cultivars, sites, growth stages, and seasons owing to the high variability of canopy reflectance signatures from one condition to another^8,15,16^. In addition, different SRIs are strongly sensitive to variations in both the structural and biochemical characteristics of the canopy^17,18^. Therefore, a full VIS-SWIR spectrum should be considered when generalizing the spectral data to estimate the crop variables under heterogeneous growing conditions.

To identify the important bands associated with the crop variables of interest within the full VIS-SWIR spectrum, multiple linear regression analysis (MLR) has been commonly used. However, a full VIS-SWIR spectrum often consists of hundreds of wavelengths that are redundant, noisy, and/or correlated by chance^19–21^. Therefore, in order to build robust spectral indices, it is necessary to isolate such wavelengths. Partial least squares regression (PLSR), which is related to both MLR and principle component regression, is an effective method for dealing with this type of data and overcomes the problems of overfitting and multicollinearity that are inherent to hyperspectral datasets by reducing such datasets into new orthogonal latent variables (OLVs). In particular, PLSR is able to correlate data when the number of independent variables greatly exceeds the number of dependent variables^22,23^. Therefore, many studies have applied PLSR to estimate various crop physiological, biochemical, nutrient, and structural parameters such as the photosynthetic capacity^24^, leaf transpiration rate^25^, leaf water potential and *Gs*^8^, pigment, nitrogen, and potassium content^9,16,26^, green and dry biomass^27,28^, leaf area index^18^, and grain yield^29–31^. Therefore, in this study, we hypothesized that the combinations of all hyperspectral data with the PLSR method could improve the estimation of crop parameters across different environmental conditions and genotypes compared with published SRIs. Hence, the main objective of this study was to test the performance of PLSR and band selection techniques that are based on the variable importance in projection (VIP) of PLSR in combination with MLR analysis in order to estimate the growth and photosynthetic efficiency of two wheat genotypes grown under different salinity levels. Tests of this objective could derive a universal model that could be applied for monitoring growth and photosynthetic-related traits under saline field conditions.

## Materials and Methods

This study was conducted under simulated close-to-field conditions using the subsurface water retention technique (SWRT) over two consecutive years (2016/2017 and 2017/2018). This technique is able to avoid the spatial and temporal heterogeneity of salt concentrations in the root zone that are common under natural saline field conditions^2,32^. In addition, this technique provides a sufficient measuring area for applying a high-throughput phenotyping tool, which is difficult to achieve with a pot experiment. The setup of SWRT is described in detail by El-Hendawy *et al*.^2,32^. The soil texture is sandy loam with a bulk density, field capacity, and wilting point of 1.48 g cm^−3^, 0.215 m^3^ m^−3^, and 0.101 m^3^ m^−3^, respectively. In the 0–40 cm soil layer, organic matter, and available N, P_2_O_5_ and K_2_O were 7.8 g kg^−1^, 45.15 mg kg^−1^, 2.443 mg kg^−1^, and 186.91 mg kg^−1^, respectively.

Two spring wheat cultivars differing in their salt tolerance [salt-sensitive Sakha 61 and salt-tolerant Sakha 93 (El-Hendawy *et al*.^33^)] were used in this study. The two cultivars were exposed to three salinity levels (control, 60, and 120 mM NaCl). The control treatment was irrigated with freshwater during all growth stages, while the treatments of 60 and 120 mM NaCl were irrigated with artificial saline water containing 3.5 L and 7.02 g NaCl L^−1^, respectively, after 25 days from sowing. A surface irrigation system consisting of a main line connected to a plastic tank (3.0 m^3^) and distributing to sub-main hoses at each plot was used. To apply an equal and constant amount of irrigation water, the main line and sub-main hoses were equipped with a water meter and a manual control valve, respectively.

The experiment was laid out in a two-factor split-plot design with three randomized complete blocks as replications. Each salinity level was split in two subplots, and the two cultivars were distributed randomly in these subplots. Each subplot size was 6.0 m long and 1.2 m wide (two SWRT membrane sheets with a thickness of 0.03 mm). The seeds were drilled in rows spaced 15 cm apart on December 5, 2016 and 2017, with a seeding rate and depth of 17 g m^−2^ and 3 cm, respectively, for each cultivar. Nitrogen (N), potassium (K), and phosphorus (P) were applied at rates of 180 N, 60 K, and 90 P kg ha^−1^, respectively. The first dose of N and entire doses of P were applied at sowing. The second dose of N and the entire dose of K were applied at the stem elongation stage. The third dose of N was applied at the booting stage.

### Plant parameter and spectral measurements

The data of plant parameters and spectral characteristics were measured at the anthesis growth stage (around 90 days from sowing). The net photosynthesis (*Pn*) rate, transpiration (*E*) rate, and stomatal conductance (*Gs*) was directly measured in the field between 09:30 and 12:00 on the second fully expanded leaf using an Li-6400 portable gas exchange system (Li-COR Inc., Biosciences, Lincoln, NE, USA). The data of the three photosynthesis parameters were collected as an average of 20 leaves for each subplot.

To determine the amount of shoot dry biomass (SDW), an area of 0.15 m^2^ was cut above ground, brought to the lab, cut into small pieces, and then desiccated in a forced-air oven at 70 °C to constant weight.

Along with measurements of growth and photosynthetic-related traits, the spectral data of canopy reflectance were collected using a portable Field Spec spectroradiometer (Analytical Spectral Devices Inc., Boulder, CO, USA). The sensors detected the reflectance from the canopy from 350 to 2500 nm in the electromagnetic spectrum at sampling intervals of 1.4 and 2.2 nm for the spectral regions from 350 to 1000 nm and from 1000 to 2500 nm, respectively. However, the final spectral data were calculated automatically to achieve a 1-nm spectral resolution. Spectral measurements were taken under sunny and windless conditions at a nadir-looking 25° angle from 80 cm above the wheat canopy to achieve a collection area of approximately 23.0 cm in diameter. The reflectance measurements were calibrated using a Spectralon white reference panel before the measurements and at every 10 min or when needed to avoid any effects generated by atmospheric conditions or sun irradiance. Five measurements were taken from each plot with an average of 10 scans for each measurement, while the final measured spectrum for each plot was an average of five sequential measurements.

### Data analysis

An analysis of variance (ANOVA) appropriate for a randomized complete block split-plot design was performed to test the response of the growth and photosynthetic-related traits of two wheat cultivars at different salinity levels. The salinity levels and wheat cultivars were considered as the main factor and the subfactor, respectively. Linear regression between the shoot dry weight and different photosynthetic-related traits was performed using Sigma Plot (Sigma Plot 11.0).

PLSR and MLR analyses were performed using the XLSTAT statistical software package (vers. 2019.1, Excel Add-ins soft SARL, New York, NY, USA).

PLSR analysis is more robust when the number of prediction variables (wavelength data, X) is larger than the number of response variables (measurement parameters, Y). The main target of PLSR analysis is to avoid underfitting or overfitting inherent in the spectral data by selecting the optimal number of latent variables (ONLVs). In the current study, the ONLVs in the PLSR were selected according to the cumulative values of Q^2^ (Q^2^ cum) and cumulative values of the coefficients of determination (R^2^) for the X and Y variables (R^2^X cum and R^2^Y cum) (Figs S1 and S2). Q^2^ represents the fraction of the total variation in Y variables that can be predicted by a given component, while Q^2^ cum indicates the fraction that can be predicted by all components. R^2^Y cum and R^2^X cum indicate the cumulative values of R^2^ for Y and X variables, respectively. In general, the ONLVs are selected when the Q^2^cum >0.5 and R^2^Y cum and R^2^X cum are close to 1.0^34^.

The performance ability of PLSR analysis based on the full spectral region was evaluated by the coefficient of determination (R^2^), root mean square error (RMSE), and relative error (RE, %) in both the calibration (Cal) and validation (Val) datasets. For each measured parameter in each condition, the PLSR models in both Cal and Val were acceptable when the models exhibited the highest values for R^2^ and the lowest values for RMSE and RE. In addition, 75% of the data set was randomly assigned to Cal data, and the remaining data set (25%) was assigned to Val data.

The effective waveband regions in the full spectrum for each parameter and condition were established under the ONLVs. These wavebands were identified based on the variable importance in the projection (VIP) derived from the PLSR analysis. The VIP values represent the relative importance of each wavelength to the PLSR model. Wavelengths with higher values of VIP indicated that these wavelengths in the PLSR model were more important than other for estimating the measured parameters. The most influential waveband regions were retained when their VIP value was greater than 1.02^22^.

To identify the most important wavelength contributing to the target parameter estimation, which is not possible in PLSR analysis, the effective waveband regions were further applied to MLR analysis as independent variables. Then, the MLR models were constructed based on these influential wavelengths, which were selected for each parameter in each condition.

## Results

### Analysis of variance

Table 1 displays a summary of the statistical analysis related to the mean square values of the analysis of variance and the mean values of treatments for combined data over two years for shoot dry weight (SDW) and photosynthetic parameters [net photosynthesis rate (*Pn*), stomatal conductance (*Gs*), and transpiration rate (*E*)] measured at anthesis. The analysis revealed that the main effects of the years, salinity levels, and cultivars were significant (*p* ≤ 0.05) for all parameters. In addition, the salinity level by cultivar interaction was also significantly different for all parameters except *E*. The interactions between the year and salinity level; year and cultivar; and year, salinity level, and cultivar showed insignificant differences for all parameters with the exception of SDW, which showed significant differences for the year by the salinity level interaction (Table 1).

**Table 1:** Statistical analysis (degrees of freedom (df), mean square value, and significance level) and mean values of the mean factor of the combined data of the shoot dry weight (SDW), photosynthetic rate (*Pn*), stomatal conductance (*Gs*), and transpiration rate (*E*) measured at the anthesis growth stage.

Source of variance	df	SDW (g m^−2^)	*Pn*(µmol CO_2_ m^−2^ s^−1^)	*Gs*(mmol m^−2^ s^−1^)	*E*(mmol m^−2^ s^−1^)
**Mean squares values**					
Year (Y)	1	282598.6^*^	17.92^*^	669.08^*^	2.27^*^
Salinity (S)	2	2706843.2^***^	209.10^***^	72986.4^***^	5.92^***^
S * Y	2	11899.8^*^	0.814^ns^	281.60^ns^	0.016^ns^
Cultivars (C)	1	592181.6^***^	154.6^***^	11130.25^***^	0.635^**^
C*Y	1	2916.0^ns^	3.60^ns^	380.25^ns^	0.041^ns^
C * S	2	6279.5^*^	3.66^*^	471.08^*^	0.012^ns^
C * S * Y	2	2041.4^ns^	1.86^ns^	68.58^ns^	0.006^ns^
Coefficient of variation (CV%)		**5.37**	**8.81**	**6.82**	**6.36**
**Mean values of mean factor ± standard deviation**					
Salinity	0 mM	1914.0 ± 177.9	17.65 ± 3.18	261.83 ± 24.10	3.86 ± 0.37
	60 mM	1268.2 ± 200.7	12.13 ± 2.37	143.23 ± 29.86	3.09 ± 0.33
	120 mM	987.9 ± 145.0	9.47 ± 1.89	114.8 ± 14.62	2.46 ± 0.35
Cultivars	Sakha 93	1518.3 ± 408.5	15.16 ± 4.20	190.87 ± 66.52	3.00 ± 0.67
	Sakha 61	1261.8 ± 420.6	11.01 ± 3.22	155.71 ± 67.81	3.27 ± 0.67

*^,^**^,^***Significant at the 0.05, 0.01 and 0.001 probability levels, respectively, and ns: not significant.

The mean values of the tested parameters significantly decreased with increasing salinity levels, and these decreases were more pronounced in the salt-sensitive cultivar Sakha 61 than in the salt-tolerant cultivar Sakha 93 (Table 1).

### Relationships between growth and photosynthetic parameters

**Figure 1:**
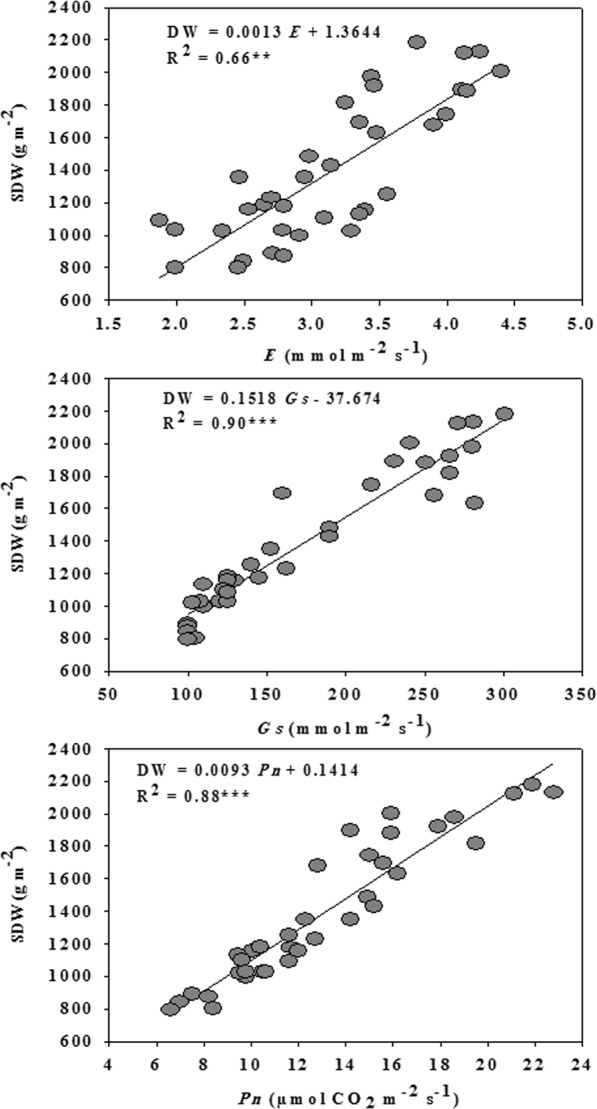
Relationship between shoot dry weight (SDW) and photosynthetic parameters [net photosynthesis rate (*Pn*), stomatal conductance (*Gs*), and transpiration rate (*E*)] for the pooled data (n = 36).

In order to explain the contribution of different photosynthetic parameters to the biomass production evaluation, linear regression analysis was performed between SDW and the three photosynthetic parameters (*Pn*, *Gs*, and *E*) using the pooled data of years, replications, salinity levels, and cultivars (Fig. 1). Generally, the three photosynthetic parameters had strong relationships with SDW, and *Pn*, *Gs*, and *E* explained 88, 90, and 66% of the variation in SDW, respectively (Fig. 1).

### Precision of growth and photosynthetic parameters evaluated by PLSR model

#### PLSR models for the estimation of the measured parameters in different conditions

The main target of PLSR analysis is to decompose dependent (measured parameters, Y) and independent (wavelengths, X) variables and find a new optimal number of latent variables (ONLVs) in order to maximize the covariance between the X and Y variables. In this study, the ONLVs were first identified based on the simultaneous highest values of Q^2^, R^2^X, and R^2^Y cumulative (Figs S1 and S2). Based on these criteria, the ONLVs were varied from 1 to 4 for SDW, *Pn*, and *Gs* and from 1 to 3 for *E* when the data were analyzed for each season, salinity level, and cultivar individually or for all of the data pooled together (Figs S1 and S2). These ONLVs established the most effective PLSR model and gave a low RMSE and RE with a high coefficient of determination (R^2^) for each parameter and condition in both the calibration and validation models (Table 2).

**Table 2:** Calibration and validation statistics of partial least square regression (PLSR) models based on the entire full wavelengths (350–2500 nm) for estimating shoot dry weight per square meter (SDW), net photosynthesis rate (*Pn*), stomatal conductance (*Gs*), and transpiration rate (*E*)) under each season (n = 18), salinity level (n = 12), and cultivar (n = 18) individually and all pooled data (n = 36). The abbreviations Treatm. and Par. indicate treatments and parameters, respectively.

Treatm.	Par.	ONLVs	Calibration dataset			Validation dataset		
			R²	RMSE_._	RE (%)	R²	RMSE_._	RE (%)
Pooled data	SDW	4	0.82^***^	181.47	13.06	0.82^***^	173.54	12.48
	*P*_*n*_	4	0.79^***^	1.93	14.75	0.79^***^	2.040	15.59
	*Gs*	4	0.81^***^	29.67	17.12	0.80^***^	28.11	16.22
	*E*	3	0.53^***^	0.455	14.51	0.53^**^	0.406	12.95
1^st^ Season	SDW	3	0.86^***^	164.52	11.13	0.86^***^	170.32	11.52
	*P*_*n*_	3	0.85^***^	1.719	12.47	0.90^***^	1.495	10.84
	*Gs*	3	0.83^***^	27.61	15.55	0.81^***^	29.72	16.73
	*E*	1	0.30^*^	0.503	14.85	0.43^*^	0.430	12.70
2^nd^ Season	SDW	3	0.79^***^	177.97	13.67	0.80^***^	168.95	12.98
	*P*_*n*_	1	0.60^**^	2.384	19.26	0.58^**^	2.296	18.55
	*Gs*	4	0.84^***^	27.61	16.34	0.85^***^	27.89	16.51
	*E*	3	0.58^**^	0.409	14.18	0.53^**^	0.408	14.14
Control	SDW	3	0.81^***^	73.72	3.85	0.85^***^	62.75	3.28
	*P*_*n*_	2	0.72^**^	1.625	9.21	0.70^***^	1.602	9.08
	*Gs*	2	0.44^*^	17.25	6.59	0.55^**^	17.23	6.58
	*E*	3	0.76^***^	0.176	4.56	0.78^***^	0.167	4.33
60 mM NaCl	SDW	1	0.64^**^	115.97	9.14	0.63^**^	128.03	10.10
	*P*_*n*_	3	0.90^***^	0.733	6.04	0.94^***^	0.514	4.24
	*Gs*	1	0.59^**^	18.21	12.71	0.62^**^	18.877	13.18
	*E*	3	0.80^***^	0.138	4.47	0.95^***^	0.077	2.49
120 mM NaCl	SDW	1	0.58^**^	89.61	9.07	0.62^**^	85.03	8.61
	*P*_*n*_	3	0.76^***^	0.822	8.68	0.74^***^	0.952	10.06
	*Gs*	2	0.53^**^	9.64	8.40	0.54^*^	9.87	8.60
	*E*	1	0.55^**^	0.223	9.07	0.66^**^	0.205	8.34
Sakha 93	SDW	3	0.84^***^	157.71	10.39	0.90^***^	124.98	8.23
	*P*_*n*_	3	0.86^***^	1.530	10.09	0.86^***^	1.393	9.19
	*Gs*	4	0.88^***^	22.73	11.91	0.87^***^	22.95	12.02
	*E*	2	0.78^***^	0.302	10.07	0.78^***^	0.250	8.33
Sakha 61	SDW	4	0.88^***^	144.14	11.42	0.92^***^	104.8	8.31
	*P*_*n*_	1	0.70^***^	1.728	15.69	0.65^**^	1.915	17.39
	*Gs*	4	0.92^***^	18.131	11.64	0.91^***^	19.044	12.23
	*E*	1	0.67^**^	0.378	11.56	0.71^***^	0.380	11.52

*^,^**^,^***Significant at the 0.05,0 0.01, and 0.001 probability levels, respectively, and ns: not significant.

Table 2 summarizes the calibration (Cal.) and validation (Val.) datasets of the PLSR models for estimating the measured parameters based on the full spectral range (350–2500 nm). The performance of the PLSR model for estimating the parameters was based on the above-mentioned conditions. The PLSR model showed good estimations of SDW, *Pn*, and *Gs* in the first season (${{\rm{R}}}_{{\rm{cal}}{\rm{.}}}^{2}$ and ${{\rm{R}}}_{{\rm{val}}{\rm{.}}}^{2}$ ranged from 0.83 to 0.86 and from 0.81 to 0.90, respectively) and SDW and *Gs* in the second season (${{\rm{R}}}_{{\rm{cal}}{\rm{.}}}^{2}$ and ${{\rm{R}}}_{{\rm{val}}{\rm{.}}}^{2}$ ranged from 0.79 to 0.84 and from 0.80 to 0.85, respectively). For the three salinity levels, the PLSR model gave a good accurate estimation of SDW, *Pn*, and *E* in the control treatment (${{\rm{R}}}_{{\rm{cal}}{\rm{.}}}^{2}$ ≥ 0.72 and ${{\rm{R}}}_{{\rm{val}}{\rm{.}}}^{2}$ ≥ 0.70), *Pn* and *E* in 60 mM NaCl (${{\rm{R}}}_{{\rm{cal}}{\rm{.}}}^{2}$ ≥ 0.80 and ${{\rm{R}}}_{{\rm{val}}{\rm{.}}}^{2}$ ≥ 0.94), and *Pn* in 120 mM NaCl (${{\rm{R}}}_{{\rm{cal}}{\rm{.}}}^{2}$ ≥ 0.76 and ${{\rm{R}}}_{{\rm{val}}{\rm{.}}}^{2}$ ≥ 0.74) (Table 2). The PLSR model provided good accurate estimations of all parameters in both the Cal. and Val. datasets for the salt-tolerant and salt-sensitive cultivars. When all data were pooled together, the PLSR model in both the Cal. and Val. datasets performed well when estimating SDW, *P*_*n*_, and *Gs*, with values of ${{\rm{R}}}_{{\rm{cal}}{\rm{.}}}^{2}$ and ${{\rm{R}}}_{{\rm{val}}{\rm{.}}}^{2}$ ≥ 0.79 (Table 2). The PLSR model showed a moderate estimation performance of SDW at 60 and 120 mM NaCl, *P*_*n*_ in the second season, *Gs* in the three salinity levels and *E* in the two seasons, 120 mM NaCl, and the pooled data, with values of R^2^_Cal._ and R^2^_val._ for all of these models ranging from 0.30 to 0.62 (Table 2).

#### Identification of optimal waveband regions through PLSR-VIP

**Figure 2:**
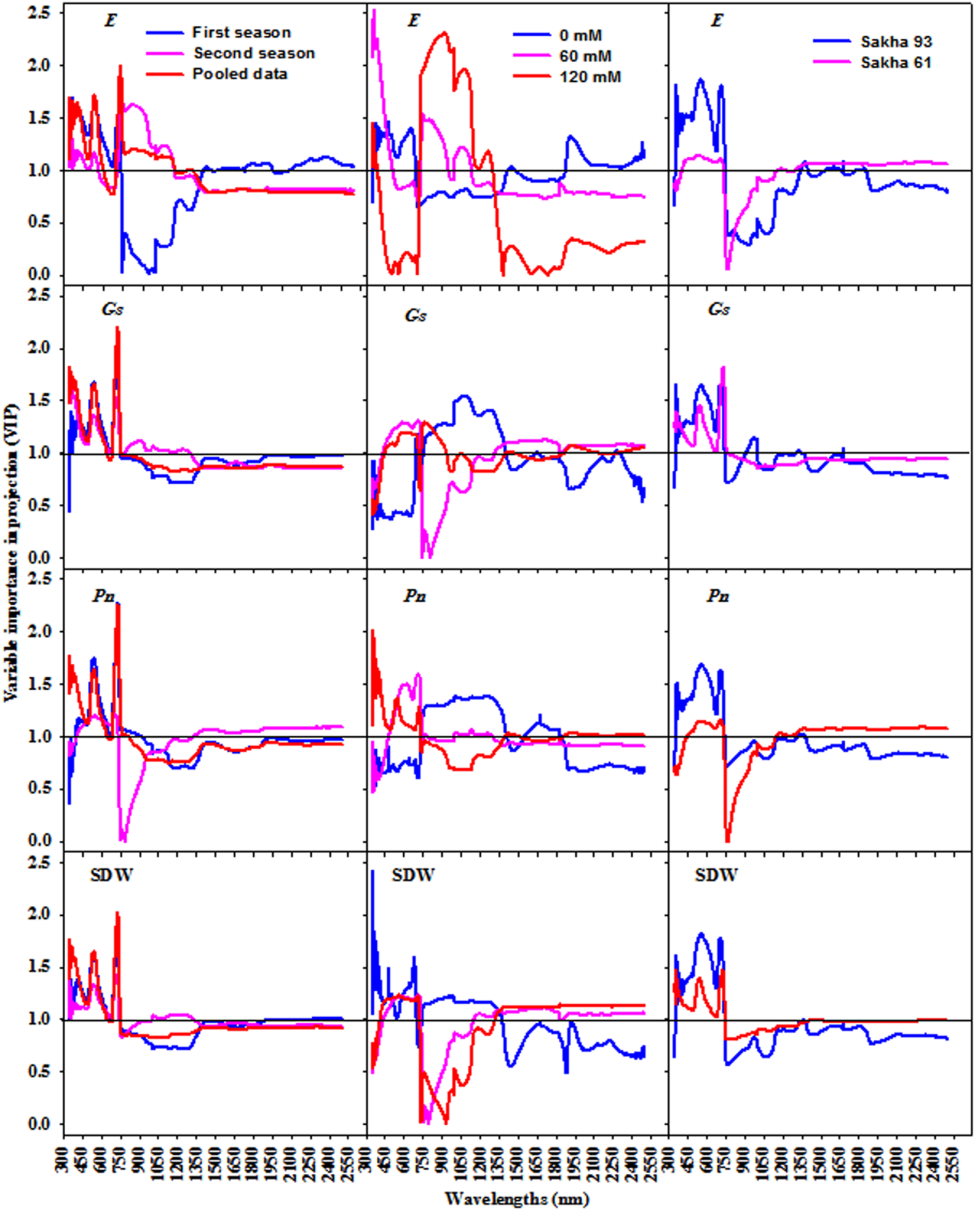
The variable importance in projection (VIP) of PLSR analysis over the entire wavelengths to extract the sensitive spectral band intervals for each measured parameter (shoot dry weight per square meter (SDW), net photosynthesis rate (*Pn*), stomatal conductance (*Gs*), and transpiration rate (*E*)) under different conditions (salinity levels, cultivars, seasons, and pooled data).

**Figure 3:**
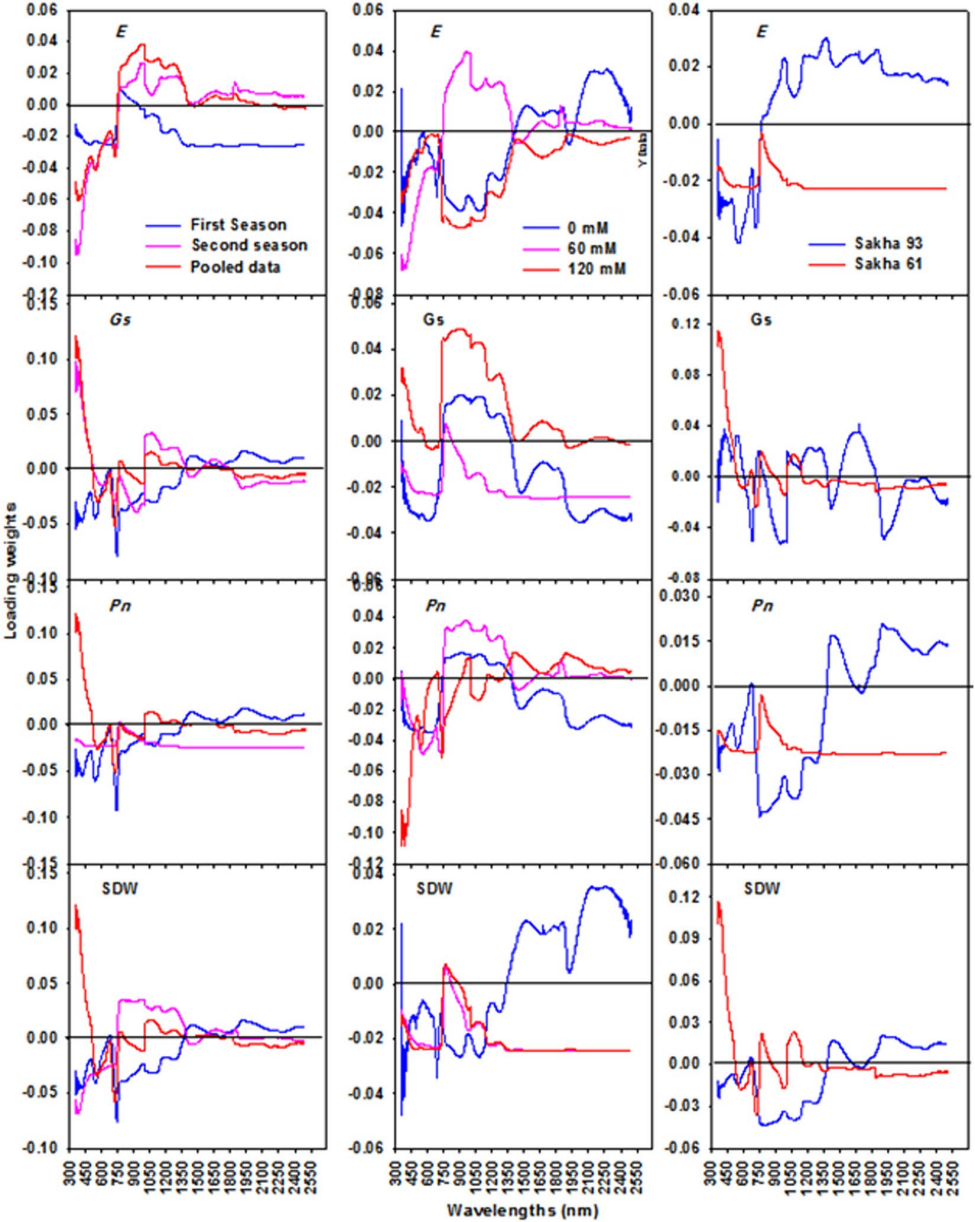
The loading weights of PLSR analysis over the entire wavelengths to extract the sensitive spectral band intervals for each measured parameter (shoot dry weight per square meter (SDW), net photosynthesis rate (*Pn*), stomatal conductance (*Gs*), and transpiration rate (*E*)) under different conditions (salinity levels, cultivars, seasons, and pooled data).

Figures 2 and 3 show the VIP scores and loading weights derived from the PLSR analysis and used to extract the important waveband regions from the full-spectrum regions (350 to 2500 nm). Generally, the important waveband regions were extracted when their VIP scores were higher than 1.0 (the threshold score) and coincided with a high absolute loading weight. These regions for each parameter in each condition are listed in Table 3. Based on the threshold score of VIP and loading weights, the visible (VIS, 350–700 nm) and red-edge (700–770 nm) spectrum were especially influential regions for all parameters in all conditions (salinity levels, cultivars, and seasons), excluding the VIS region for *P*_*n*_ and *Gs*, and the red edge for *E* in the control treatment (Table 3).

**Table 3:** Extraction of the important sensitive spectral band intervals based on the variable importance in projection (VIP) and loading weights of PLSR analysis over the entire wavelengths and the most influential wavelengths using the stepwise multiple linear regression for shoot dry weight per square meter (SDW), net photosynthesis rate (*Pn*), stomatal conductance (*Gs*), and transpiration rate (*E*)) under each season (n = 18), salinity level (n = 12), and variety (n = 18) individually and all pooled data (n = 36).

Treatm.	Par.	Spectral band intervals	Influential wavelength	R^2^	RMSE	AIC
Pooled data	SDW	350–651	450	0.78^***^	203.7	384.7
		690–752	720	0.77^***^	207.5	386.1
	*P*_*n*_	350–651	533	0.69^***^	2.40	64.95
		689–817	715, 719, 817	0.77^***^	2.11	57.57
	*Gs*	350–647	481, 548	0.77^***^	33.9	256.5
		690–760	721	0.74^***^	35.5	258.9
	*E*	350–606	536, 605	0.56^**^	0.459	−53.13
		699–1149	734	0.53^**^	0.469	−52.50
		1252–1273	1273	0.26^*^	0.587	−36.44
1^st^ Season	SDW	350–751	540	0.86^***^	174.8	187.8
	*P*_*n*_	402–916	533, 903	0.85^***^	1.88	25.46
	*Gs*	350–651	533	0.80^***^	31.8	126.4
	*E*	350–740	368, 382	0.74^***^	0.334	−36.81
		1356–1413	—	—	—	—
		1486–1889	1830	0.23^*^	0.560	−19.02
		2023–2500	2300	0.26^*^	0.550	−19.62
2^nd^ Season	SDW	350–747	724	0.81^***^	178.6	188.5
		1001–1330	1330	0.54^**^	276.3	204.2
	*P*_*n*_	407–735	716	0.72^***^	2.12	28.88
		1336–2500	2395, 2452, 2493	0.80^***^	1.92	26.95
	*Gs*	350–758	727	0.79^***^	33.6	128.4
		788–1308	1138	0.47^*^	53.1	144.9
	*E*	350–570	537, 570	0.61^***^	0.483	−23.46
		712–1141	738	0.55^**^	0.449	−26.95
Control	SDW	350–702	356, 358	0.87^***^	71.2	104.9
		744–1337	—	—	—	—
	*P*_*n*_	741–1415	1252	0.69^***^	1.87	16.83
		1557–1848	1680, 1830, 1848	0.94^***^	0.92	1.26
	*Gs*	696–728	—	—	—	—
		756–1407	760, 1078	0.73^***^	13.8	65.6
		2266–2312	—	—	—	—
	*E*	355–695	482	0.70^***^	0.215	−35.08
		1426–1485	1452	0.48^**^	0.283	−28.51
		1878–2500	1904	0.67^***^	0.226	−33.86
60 mM NaCl	SDW	440–737	722	0.73^***^	108.8	114.4
		1153–1830	1709	0.59^**^	134.5	119.5
		1861–2500	2488, 2492, 2497	0.88^***^	82.2	109.0
	*P*_*n*_	483–744	723	0.79^***^	1.14	5.06
		761–784	—	—	—	—
		1001–1305	1198	0.53^**^	1.71	14.66
	*Gs*	435–737	720	0.77^***^	14.9	66.7
		1319–2500	1710, 1831	0.76^***^	16.2	69.4
	*E*	350–514	—	—	—	—
		735–953	762	0.35^*^	0.275	−29.15
		1001–1136	—	—	—	—
120 mM NaCl	SDW	413–725	566	0.68^***^	86.2	108.8
		1321–2500	1386, 1547, 1832	0.88^***^	59.6	101.2
	*P*_*n*_	350–733	569	0.68^***^	1.13	4.64
		1396–1501	1489	0.53^**^	1.35	9.06
		1869–2500	2492, 2500	0.75^***^	1.05	3.82
	*Gs*	449–716	598	0.61^***^	10.7	58.8
		739–937	—	—	—	—
		1412–1471	1435	0.38^**^	12.1	61.7
		1866–2123	1927	0.42^**^	11.6	60.7
		2309–2500	2448, 2500	0.64^***^	9.7	57.1
	*E*	350–389	—	—	—	—
		727–1303	924	0.57^**^	0.239	–32.58
Sakha 93	SDW	353–744	578	0.76^***^	205.4	193.6
	*P*_*n*_	353–743	579	0.72^***^	2.28	31.59
		1322–1381	—	—	—	—
	*Gs*	353–744	573	0.66^***^	40.3	134.9
		920–1000	—	—	—	—
		1310–1354	—	—	—	—
	*E*	353–747	569	0.64^***^	0.413	–29.95
		1346–1402	—	—	—	—
		1540–1666	—	—	—	—
		1818–1856	—	—	—	—
Sakha 61	SDW	350–747	547	0.87^***^	156.6	183.8
	*P*_*n*_	450–737	714	0.80^***^	1.48	16.06
		1149–2500	2309	0.71^***^	1.80	23.06
	*Gs*	350–670	468, 555	0.90^***^	22.7	115.1
		678–770	717	0.88^***^	24.0	116.3
	*E*	408–736	532	0.75^***^	0.349	−36.01
		1162–1215	1191, 1215	0.71^***^	0.386	−31.58
		1304–2500	2300	0.66^***^	0.402	−30.95

*^,^**^,^***Significant at the 0.05,0 0.01, and 0.001 probability levels, respectively, and ns: not significant.

The near-infrared (NIR, 770–1300 nm) spectrum was identified as an unimportant region for SDW in 120 mM NaCl, two cultivars, the first season, and all pooled data; *P*_*n*_ in 120 mM NaCl, the cultivar Sakha 93, and the second season; *Gs* in 60 mM NaCl, the cultivar Sakha 61, first season, and all pooled data; and *E* in the control treatment, the cultivar Sakha 93, and the first season. The shortwave infrared (SWIR, >1300 nm) spectrum appeared to be an unimportant region for SDW in the control treatment, two cultivars, the first season, and all pooled data; *P*_*n*_ in 60 mM NaCl, the first season, and all pooled data; *Gs* for the cultivar Sakha 61, two seasons, and all pooled data; and *E* in 60 and 120 mM NaCl, the second season, and all pooled data (Table 3).

#### Identification of influential wavelengths through multiple linear regression (MLR)

The important waveband regions that were selected based on PLSR-VIP and loading weights were further used as independent variables in MLR in order to extract the most influential wavelengths for each parameter in each condition (Table 3). Generally, the influential wavelengths selected for each parameter are largely dependent on the conditions. The wavelengths extracted from VIS and the red edge had highly significant contributions for estimating SDW, *P*_*n*_, and *Gs* in most conditions, with R^2^ ranging from 0.61 to 0.90. The wavelengths extracted from the VIS spectrum were informative for *E* in two seasons, the control treatment, and both cultivars, with R^2^ ranging from 0.61 to 0.75. The wavelengths extracted from the red-edge spectrum had moderately significant contributions for the estimation of *E* in all pooled data, the second season, and 60 mM NaCl, with R^2^ ranging from 0.35 to 0.55 (Table 3). Note also that when using MLR, no wavelengths extracted from the NIR spectrum are important for the estimation of SDW in all conditions, as well as all parameters in the first season and the cultivar Sakha 93. In addition, no wavelengths extracted from the SWIR spectrum are important for the estimation of all parameters in all pooled data and for Sakha 93. The wavelengths extracted from the SWIR spectrum had highly significant contributions for the estimation of SDW and *Gs* in 60 and 120 mM NaCl; *P*_*n*_ in the second season, control, 120 mM NaCl, and Sakha 61; and *E* in the control and Sakha 61, with R^2^ ranging from 0.64 to 0.94 (Table 3).

Generally, irrespective of the conditions, the most influential wavelengths extracted from the VIS spectrum were found at 356, 358, 450, 540, 547, 566, and 578 nm for SDW; 533, 569, and 579 nm for *P*_*n*_; 468, 481, 533, 548, 555, 573, and 598 nm for *Gs*; and 368, 382, 482, 532, 536, 537, 569, 570, and 605 nm for *E*. The wavelengths extracted from the red-edge spectrum were found at 720, 722, and 724 nm for SDW; 714, 715, 716, 719, and 723 nm for *P*_*n*_; 717, 720, 721, 727, and 760 nm for *Gs*; and 734, 738, and 762 nm for *E*. The wavelengths at 817, 1198, and 1252 nm; 1078 and 1138 nm; and 924, 1191, 1215, and 1273 nm for *P*_*n*_, *Gs*, and *E*, respectively, were extracted from the NIR spectrum as the most influential wavelengths. The most influential wavelengths extracted from the SWIR spectrum were found at 1330, 1386, 1547, 1709, 1832, 2488, 2492, and 2497 nm for SDW; 1489, 1680, 1830, 1848, 2309, 2395, 2452, 2492, 2493, and 2500 nm for *P*_*n*_; 1435, 1710, 1831, 1927, 2448, and 2500 nm for *Gs*; and 1452, 1830, 1904, and 2300 nm for *E* (Table 3).

#### Prediction of the measured parameters based on the full spectrum using a PLSR model

**Figure 4:**
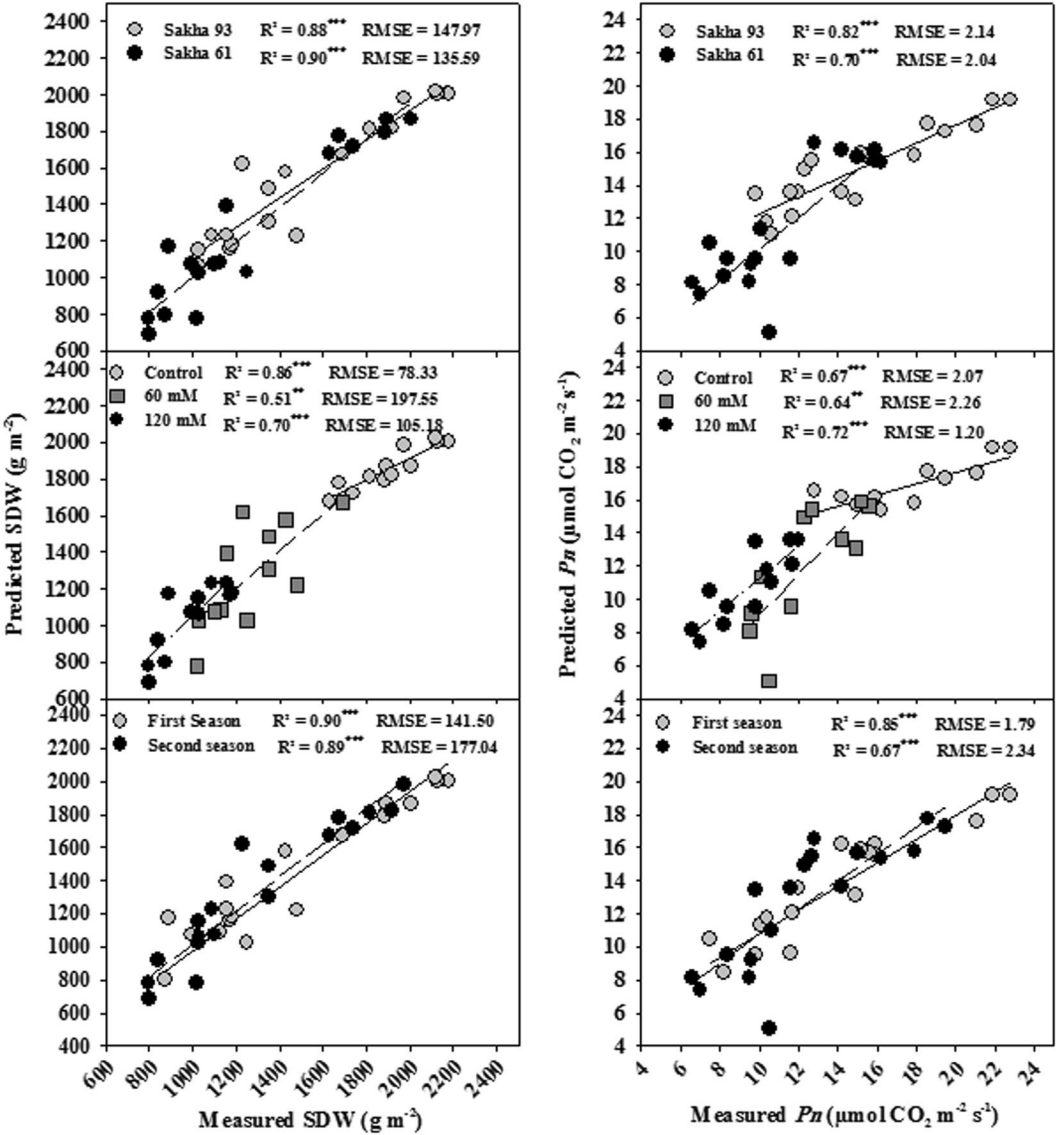
Relationship between the observed and cross-validated prediction values of shoot dry weight per square meter (SDW) and net photosynthesis rate (*Pn*) under different conditions (salinity levels, cultivars, seasons) as predicted by the PLSR model.

**Figure 5:**
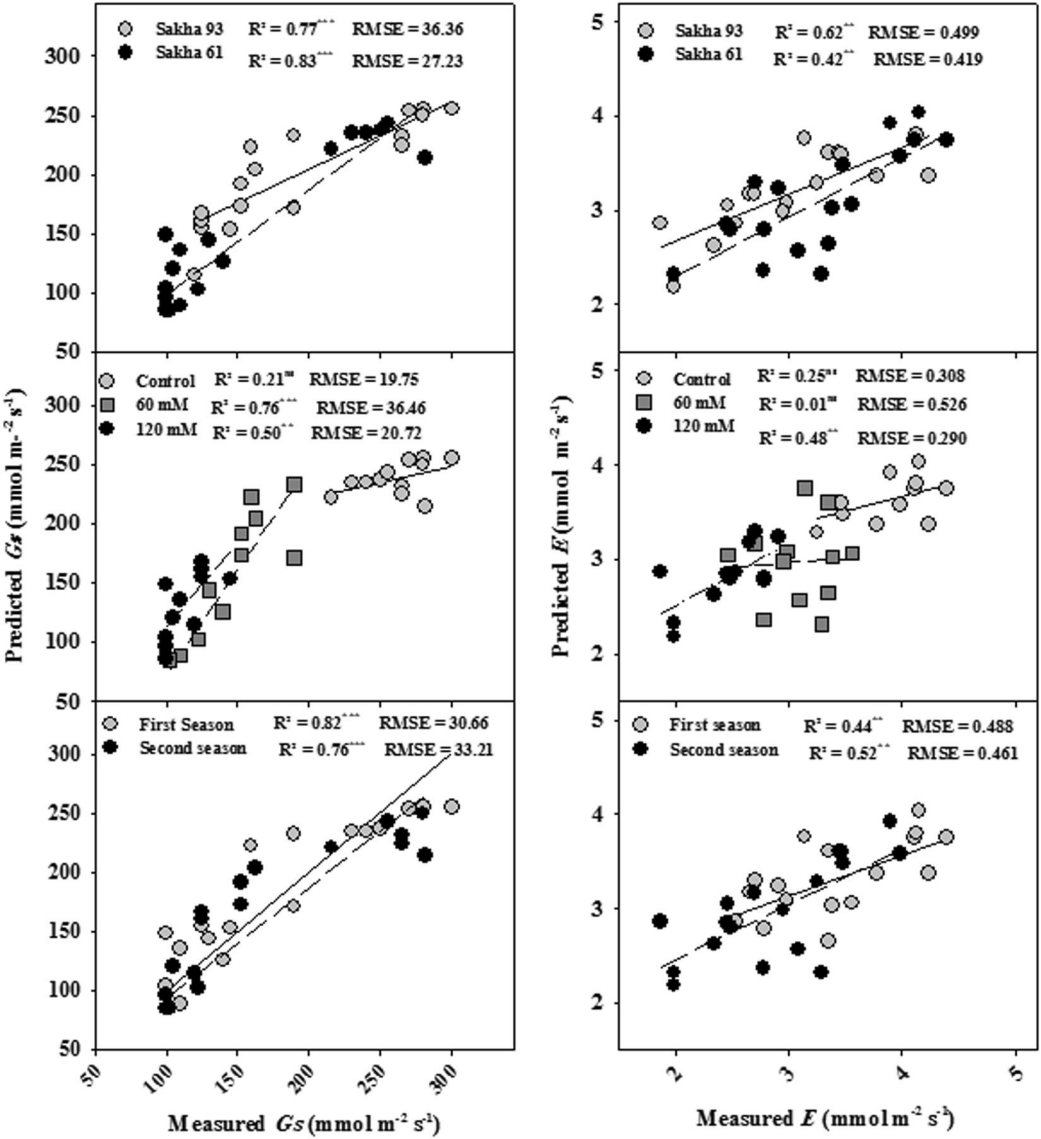
Relationship between the observed and cross-validated prediction values of stomatal conductance (*Gs*) and transpiration rate (*E*) under different conditions (salinity levels, cultivars, seasons) as predicted by the PLSR model.

Figures 4 and 5 show the relationship between the observed and cross-validated prediction values of the measured parameters in each condition as predicted by the PLSR model. In general, the PLSR models provided a more accurate estimation of SDW, *P*_*n*_, and *Gs* than of *E* in all conditions (Figs 4 and 5). The predictive ability was strongest for SDW in two seasons, two cultivars, and the control treatment (R^2^ = 0.86–0.90); *P*_*n*_ in the first season and Sakha 93 (R^2^ = 0.85 and 0.82, respectively); and *Gs* in two seasons, two cultivars, and 60 mM NaCl (R^2^ = 0.76–0.83). The predictive ability was good for SDW in 120 mM NaCl (R^2^ = 0.70) and *P*_*n*_ in the second season, 60 and 120 mM NaCl, and Sakha 61 (R^2^ = 0.67–0.72); moderate for SDW and *P*_*n*_ in 60 mM NaCl (R^2^ = 0.51 and 0.64, respectively), *Gs* in 120 mM NaCl (R^2^ = 0.50), and *E* in two seasons, two cultivars, and 120 mM NaCl (R^2^ = 0.42–0.62); and insignificant for *Gs* and *E* in the control treatment and *E* in 60 mM NaCl (Figs 4 and 5).

## Discussion

The different components of the salinity stress (i.e., specific ion toxicities, ion imbalance, and physiological drought stress) interact to constrain all of the physiological processes that are important for plant growth and development. Under salinity conditions, a decrease in leaf turgor pressure along with a K^+^ deficit leads to dramatic changes in the stomatal conductance (*Gs*), which ultimately leads to trouble in the photosynthesis (*P*_*n*_) and transpiration (*E*) rates^2,35,36^. Additionally, it is possible to detect the negative impacts of the salinity stress on the photosynthetic capacity before damage from irreversible morphological characteristics can be detected^37^. Therefore, it is important to develop wheat cultivars with better physiological performance under salinity stress by considering the photosynthesis-related traits as screening criteria in breeding programs.

The results of this study showed that the SDW at the anthesis growth stage had a strong relationship with *P*_*n*_ (R^2^ = 0.88, p < 0.01) and *Gs* (R^2^ = 0.90, p < 0.01), and a moderate relationship with *E* (R^2^ = 0.66, p < 0.05) (Fig. 1). These results indicate that the photosynthetic-related parameters could serve as key indicators for providing valuable information about the actual salinity stress level and the status of plant physiological performance under salinity stress. Furthermore, these results also confirm that the biomass accumulation under salinity could serve as a key indicator for many physiological processes at the entire plant level. Therefore, simultaneously monitoring and assessing these traits using a nondestructive, fast, and efficient tool is an urgent task, especially in wheat genetics and breeding programs where the salt tolerance of a large number of genotypes must be evaluated every year at different stages of the plant life cycle. In this study, the canopy hyperspectral reflectance data combined with multivariate analyses (PLSR and MLR models) was used to assess variations in growth and the photosynthetic efficiency of wheat under different conditions (salinity levels, cultivars, and seasons).

### Ability of PLSR to assess variations in the measured parameters

Analysis of hyperspectral reflectance data using an appropriate statistical procedure is still a critical step in unraveling the relationship between these data and specific crop variables^38,39^. A PLSR analysis is one of the most efficient statistical procedures to determine the appropriateness of this relationship. The advantages of this analysis are that it utilizes the full relevant spectrum information, which the popular spectral reflectance indices leave out, and has the ability to effectively address the strong noise, multicollinearity, and overfitting that are inherent to hyperspectral reflectance data^21,40,41^. However, the robustness of PLSR models strongly depends on the ability to select the ONLVs; which is important in avoiding overfitting problems related to spectral data. In this study, the ONLVs in the PLSR models ranged between 1 and 4 and were considered to be effective in avoiding the overfitting problem (Table 2 and Figs S1 and S2). Similar results were reported by Maimaitiyiming *et al*.^14^ for estimating the photosynthesis-related traits (*P*_*n*_, *Gs*, and *E*) in grapevine under different levels of water stress, where the best PLSR models were also found with three ONLVs.

Furthermore, PLSR models with ONLVs ranging from 1 to 3 have been reported for assessing the growth and yield of winter wheat under different nitrogen levels^42,43^ using proximal hyperspectral data. On the other hand, PLSR models with ONLVs of 11, 15, and 21 were mentioned for assessing *P*_*n*_, *Gs*, and the leaf dry mass per area (LMA), respectively, in different elite and landrace wheat genotypes under different nitrogen levels^44^. These differences in the range of ONLVs between studies could be a result of the changing environmental conditions of the hyperspectral reflectance measurements.

Although the parameters related to the photosynthetic efficiency have gained importance when evaluating the salt tolerance of wheat genotypes, estimation of these parameters using PLSR analysis for the full spectrum range (350–2500 nm) has been less studied. Most of the literature has focused on the performance of spectral reflectance indices (SRIs) for estimating these parameters under different abiotic stresses^38,39,44–46^. Under different levels of soil water conditions in olive orchards, Lobos *et al*.^45^ reported that the PLSR model based on the full spectrum exhibited accurate estimations at the branch level in both the calibration (Cal.) and validation (Val.) datasets for *P*_*n*_ (${{\rm{R}}}_{{\rm{cal}}}^{2}$ = 0.79 and ${{\rm{R}}}_{{\rm{val}}}^{2}$ = 0.81), *Gs*, (${{\rm{R}}}_{{\rm{cal}}}^{2}$ = 0.61 and ${{\rm{R}}}_{{\rm{val}}}^{2}$ = 0.78), and *E* (${{\rm{R}}}_{{\rm{cal}}}^{2}$ = 0.65 and ${{\rm{R}}}_{{\rm{val}}}^{2}$ = 0.81). However, when the data were compared between low and high levels of soil water conditions, only *P*_*n*_ had a high R^2^ in both the Cal. and Val. datasets under a low level of water stress, while the three parameters showed a high R^2^ in Cal. (0.65 to 0.86) and medium (0.37 to 0.41) in Val. under a high level of water stress. In wheat under different nitrogen levels Silva-Perez *et al*.^44^ and blueberry under contrasting water supply and heat conditions, Lobos *et al*.^39^ described moderate to weak R^2^ for *P*_*n*_, *Gs*, and *E* (${{\rm{R}}}_{\mathrm{cal}/\mathrm{val}}^{2}$ ranging from 0.22 to 0.44). In addition, Lobos *et al*.^39^ also reported that none of the PLSR models based on the full spectrum were able to reach a ${{\rm{R}}}_{{\rm{cal}}}^{2}$ or R^2^_Val_ higher than 0.44 for three photosynthetic parameters. When we examined our data, for the four parameters evaluated (SDW, *P*_*n*_, *Gs*, and *E*), the results showed that the PLSR models based on the full spectrum generated a moderate to high R^2^ in both the Cal. and Val. datasets (R^2^ ranged from 0.30 to 0.92 in Cal. and from 0.43 to 0.95 in Val.), but this range of R^2^ values is highly dependent on the measured parameters and conditions. Only *E* in the first season recorded the lowest values of R^2^ in both the Cal. (0.30) and Val. (0.43) datasets (Table 2). These results once again confirm that the PLSR models based on the full spectrum can provide additional improvements in the accurate estimation of the measured parameters under salinity. This is because the PLSR models include a wide range of sensitive wavelengths that can cover all of the main physiological changes of plants induced by salt stress. Therefore, PLSR models based on the full spectrum provide strong performance in estimating the four measured parameters in different conditions, with few exceptions.

When comparing the observed values of the measured parameters against those predicted by the PLSR models using the full spectrum, SDW, *P*_*n*_, and *Gs* all had a higher R^2^ than *E* in all conditions, with the exception of *Gs*, which showed an insignificant relationship between the observed and predicted values under the control treatment such as *E*. The predicted values of SDW, *P*_*n*_, *Gs*, and *E* varied from 0.51 to 0.90, 0.64 to 0.85, 0.21 to 0.83, and 0.01 to 0.62, respectively (Figs 4 and 5). These findings reaffirm that by modeling the canopy spectral reflectance using PLSR, it is possible to assess growth and some key physiological parameters that could be incorporated into breeding programs oriented to improving the salt tolerance of wheat genotypes in a fast and nondestructive manner. These findings are promising because most photosynthesis-related parameters (*P*_*n*_ and *Gs*) and growth (SDW), which were previously reported as efficient screening criteria for discriminating wheat genotypes for salt tolerance^2^, were predicted with relatively high R^2^ across different salinity levels and wheat genotypes. These results are similar to those reported by Doughty *et al*.^47^ for a tropical forest, regarding the ability of the PLSR model to best predict the LMA (R^2^ = 0.90) and *P*_*n*_ (R^2^ = 0.74) followed by *E* (R^2^ = 0.48). However, for wheat, Silva-Perez *et al*.^44^ reported that the ability of PLSR to predict LMA was generally high (R^2^ = 0.90), while it was moderate for *P*_*n*_ (R^2^ = 0.49) and *Gs* (R^2^ = 0.34).

### Assessment of the measured parameters based on the combination of PLSR-VIP and MLR

Several studies reported that the importance of PLSR lies in identifying the very best waveband regions from the full spectrum which are directly and indirectly linked to the measured parameters^8,12,16,43,48,49^. The importance in projection (VIP) is a main factor in the PLSR model and provides insight into the importance of each wavelength in the full spectrum when removing wavelengths with low predictive power. In this study, the most important waveband regions for estimating all measured parameters in all conditions with VIP values higher than 1 and a high absolute loading weight were mainly located in the visible and red-edge regions with few exceptions. The NIR and SWIR regions were also identified as most important regions for estimating some measured parameters under particular conditions (Table 3). These results are somewhat similar to those of Doughty *et al*.^47^, who reported that the most important regions selected from the full spectrum for the assessment of photosynthesis-related traits in a tropical forest were located in the VIS and red-edge regions. Carter^50^ reported that the red-edge region around 710 nm is the waveband most suitable for the estimation of *P*_*n*_ and *Gs* in pine. In addition, Maimaitiyiming *et al*.^14^ found that the VIP values indicated the yellow (580–640 nm) band as the most important region for *Gs* estimation under different levels of water stress. The PLSR-VIP values revealed that the 530–550, 700–750, 1380–1420, and 1450–1590 nm bands were effective in estimating different physiological traits (leaf water potential, *Gs*, and non-photochemical quenching) in grapevine under water stress^8^.

Generally, by reviewing the wavebands selected based on PLSR-VIP and loading weights in our study (Figs 2, 3 and Table 3), it was found that many of these bands are known to be very sensitive to variations in leaf carotenoids, xanthophylls, chlorophyll pigments, fluorescence, internal structure, biomass, and water content. These are all directly associated with above-ground biomass and photosynthesis efficiency^8,14,24,44,51^. Therefore, our results indicate that the PLSR-VIP and loading weights methods can potentially identify the important waveband regions related to the growth and photosynthesis-related traits of wheat under salinity stress conditions.

The literature suggests that the ability of the full spectrum in the estimation of the measured parameters can be refined and the results can be easily interpreted through a combination of the PLSR-VIP and MLR methods. The former avoids the overfitting and multicollinearity inherent in spectral data and selecting the significant waveband regions, while the latter selects the most influential wavelengths in the final model^39,42,52^. In this study, the significant waveband regions that were identified by PLSR-VIP were applied to MLR as independent variables to identify the most influential wavelengths linked to each measured parameter in each condition. The results of the MLR analysis demonstrated that the individual or combination of two or three wavelengths selected form the VIS, red-edge, and middle NIR waveband regions accounted for most variations in SDW, *P*_*n*_, and *Gs* in all conditions. The selected wavelengths explained from 61 to 90% of the variability found in the three parameters, and the range of variability depended on the measured parameter and condition (Table 3).

The wavelengths selected from the three regions (VIS, red edge, and middle NIR) exhibited moderate to high variability in *E* (R^2^ values ranged from 0.35 to 0.75), and this variability also depended on the conditions. Interestingly, the results of the MLR analysis also demonstrated that most of the variability in the four measured parameters can be detected by a combination of two or three wavelengths selected from the SWIR region. The wavelengths selected from the SWIR region explained 88%, 71 to 94%, 64 to 76%, and 66 to 71% of the variability in SDW, *P*_*n*_, *Gs*, and *E*, respectively, and most of these variabilities were detected in the three salinity levels and for the cultivar Sakha 61 (Table 3). Generally, these results indicate that the MLR model based on the important wavelengths can be successfully used to estimate the growth and photosynthetic-related traits under salinity stress conditions, as the most influential wavelengths were retained in the model. These results confirmed findings by Sharabian *et al*.^42^ and Wang *et al*.^43^, who reported that the MLR model could be effectively used for rapidly and accurately estimating the growth status and yield of winter wheat under different levels of nitrogen treatment.

As expected, the osmotic and ionic stresses, as well as the deficit in essential ions that is imposed by salinity, resulted in considerable disturbances in the photosynthetic pigments and potential, leaf structural properties, and water content of the leaf. This ultimately leads to substantial variations in the canopy spectral reflectance in the wavelength range of VIS, red edge, NIR, and SWIR^12,43,53^. The wavelengths in the blue, green, red, and red-edge regions of the spectrum associated well with the considerable changes that occurred in the leaf pigment content and photosynthetic efficiency^31,46^. The wavelengths within the NIR region were influenced by several structural properties of the leaves and canopy, whereas the wavelengths within SWIR were always sensitive to the plant water status and leaf biochemical compounds such as cellulose, lignin, and proteins^20,43,54^. Based on the abovementioned relationship between the plant characteristics and the wavelengths within the different regions of the spectrum, logically, the SDW and photosynthetic-related traits can be successfully estimated through several wavelengths within the VIS, red-edge, and middle NIR regions. This statement was also confirmed by the results of the current study. This could explain why most of the published spectral reflectance indices developed for biomass and photosynthetic variable estimation are always formulated from wavelengths selected from each VIS, red-edge, or NIR regions or combined between the three regions^14,31,46,55^.

On the other hand, the results of the current study also showed wavelengths within the SWIR region that were found to be of some importance in the estimation of the photosynthetic-related traits (Table 3), although no wavelengths within SWIR (1900–2500 nm) were directly related to the photosynthetic efficiency. The contribution of some wavelengths within this region may be attributed to the effects of salinity stress on the water status and leaf biochemical compounds, which ultimately strongly affect the photosynthetic efficiency and capacity. Therefore, we assume that the wavelengths corresponding to the leaf water content and biochemical compounds carry important information about the photosynthetic efficiency and capacity under salinity stress conditions. Similar results were reported by Inoue *et al*.^55^ and Rapaport *et al*.^8^, who stated that the selected wavelengths within SWIR (e.g., 1330 and 1500 nm), which are almost independent of the variations in pigment content and photosynthetic capacity, were indicative of stress-induced alterations in several photosynthetic-related traits. Wang and Jin^25^ also reported that wavelengths selected by PLSR-VIP and MLR within the SWIR domain, especially at 2435, 2440, 2445, and 2470 nm, were found to be effective for tracking changes in *E* under drought stress conditions.

## Conclusion

The results of the current study indicated that by combining hyperspectral reflectance data with an appropriate statistical analysis, it is possible to accurately, rapidly, and nondestructively assess the growth- and photosynthetic-related traits of wheat under salinity stress. Combining PLSR-VIP with MLR has the potential to identify the most effective wavebands and influential wavelengths related to the growth and photosynthesis-related traits of wheat under different conditions (salinity levels, cultivars, and seasons). Based on PLSR-VIP analysis, the most important waveband regions for estimating all measured parameters in all conditions were mainly located in the visible and red-edge regions with few exceptions. The NIR and SWIR regions were also identified as most important regions for estimating some measured parameters under particular conditions. The results of the MLR analysis demonstrated that the individual or combination of two or three wavelengths selected form the VIS, red-edge, and middle NIR waveband regions accounted for most variations in SDW, *P*_*n*_, and *Gs* in all conditions (explained from 61 to 90% of the variability found in the three parameters). The results of the MLR analysis also demonstrated that most of the variability in the four measured parameters can be detected by a combination of two or three wavelengths selected from the SWIR region (explained from 64 to 94% of the variability found in the four measured parameters).

## Supplementary information

Supplementary Figure
