# Supplementary material for: Estimating growth and photosynthetic properties of wheat grown in simulated saline field conditions using hyperspectral reflectance sensing and multivariate analysis
Source: Sci Rep. 2019 Nov 11;9:16473. doi: 10.1038/s41598-019-52802-5 (PMC6848100; doi:10.1038/s41598-019-52802-5)
Supplement: Supplementary file 1 — Supplementary Figure [file 41598_2019_52802_MOESM1_ESM.doc]

**Estimating growth and photosynthetic properties of wheat grown in simulated saline field conditions using hyperspectral reflectance sensing and multivariate analysis**

**Salah El-Hendawy^1,2^,*, Nasser Al-Suhaibani^1^, Majed Alotaibi^1^, Wael Hassan^3,4^, Salah Elsayed^5^, Muhammad Usman Tahir^1^, Ahmed Ibrahim** **Mohamed^6^, Urs Schmidhalter^7^**

^1^Department of Plant Production, College of Food and Agriculture Sciences, King Saud University, P.O. Box 2460, 11451 Riyadh, Saudi Arabia

^2^Department of Agronomy, Faculty of Agriculture, Suez Canal University, Ismailia, 41522, Egypt

^3^Department of Agricultural Botany, Faculty of Agriculture, Suez Canal University, Ismailia, 41522, Egypt

^4^Department of Biology, College of Science and Humanities at Quwayiah, Shaqra University, Riyadh 11961, Saudi Arabia

^5^Evaluation of Natural Resources Department, Environmental Studies and Research Institute, University of Sadat City, Menoufia 32897, Egypt

^6^Department of Soil and Water, Faculty of Agriculture, Suez Canal University, Ismailia, 41522, Egypt

^7^Department of Plant Science, Chair of Plant Nutrition, Technical University of Munich, Freising, Germany

*** Corresponding author**

[**mosalah@ksu.edu.sa**](mailto:mosalah@ksu.edu.sa)**)**

**Supplementary Figure S1. Model quality by number of components for shoot dry weight (SDW) and photosynthetic rate (*Pn*) under different conditions (salinity levels, cultivars, seasons, and pooled data).**

**Supplementary Figure S2. Model quality by number of components for stomatal conductance (*Gs*), and transpiration rate (*E*) under different conditions (salinity levels, cultivars, seasons, and pooled data).**
